# Animal Protein Intake Is Positively Associated with Metabolic Syndrome Risk Factors in Middle-Aged Korean Men

**DOI:** 10.3390/nu12113415

**Published:** 2020-11-06

**Authors:** Sangwon Chung, Min-Yu Chung, Hyo-Kyoung Choi, Jae Ho Park, Jin-Taek Hwang, Hyojee Joung

**Affiliations:** 1 Research Group of Healthcare, Korea Food Research Institute, Jeollabuk-do 55365, Korea; schung@kfri.re.kr (S.C.); mic07002@kfri.re.kr (M.-Y.C.); chkyoung@kfri.re.kr (H.-K.C.); jaehopark@kfri.re.kr (J.H.P.); jthwang@kfri.re.kr (J.-T.H.); 2 Department of Public Health, Graduate School of Public Health, Seoul National University, Seoul 08826, Korea; 3 Department of Food Biotechnology, University of Science and Technology, Daejeon 34113, Korea; 4 Institute of Health and Environment, Seoul National University, Seoul 08826, Korea

**Keywords:** abdominal obesity, animal protein, protein intake, metabolic syndrome

## Abstract

Few studies have examined the relationship of protein intake by food source with metabolic syndrome in Korean adults, even though animal food intake has increased. This study examined the association between plant and animal protein intake and metabolic syndrome among middle-aged Korean adults. A total of 13,485 subjects aged 30–64 years were selected from the 2013–2018 Korea National Health and Nutrition Examination Survey. Protein intake was assessed using 24-h dietary recall data and divided into quintiles. Men had a higher percentage of energy intake from animal protein (7.4%) than plant protein (6.9%). Men in the highest quintile group of animal protein intake had a higher prevalence of abdominal obesity (OR: 1.30, 95% CI: 1.00–1.70), reduced high-density lipoprotein cholesterol (HDL-C) (OR: 1.43, 95% CI: 1.07–1.90), and elevated fasting glucose (OR: 1.32, 95% CI: 1.01–1.74), after adjusting for covariates. Furthermore, stronger associations of animal protein intake with abdominal obesity were shown in men who consumed less than estimated energy requirements (OR: 1.60, 95% CI: 1.11–2.31). Plant protein intake was negatively associated with increased blood pressure in men. Neither animal nor plant protein intakes were significantly associated with any of the metabolic syndrome risk factors in women. The results imply that lower animal protein intake may be a beneficial factor for metabolic syndrome management in middle-aged Korean men.

## 1. Introduction

Metabolic syndrome, defined as comorbidities of metabolic abnormalities including dyslipidemia, high blood pressure, glucose intolerance, and abdominal obesity, is a public health concern due to its link to increased risks of diabetes, cardiovascular disease, and all-cause mortality [1,2,3]. Various strategies including lifestyle modification such as a healthy diet are recommended to manage metabolic syndrome and prevent other chronic diseases [4]. Macronutrient composition is a major factor in determining a healthy diet which is known to be beneficial for weight loss and metabolic risk factors [5,6,7]. It has also been reported that dietary protein content is an important factor affecting the glycemic response related to glucose metabolism [8]; however, the role of dietary protein in the risk of metabolic disease remains inconclusive.

Diets high in protein have proved helpful for weight loss, with increased satiety and decreased energy intake, and improved cardiovascular markers including lipid profiles and blood pressure [9,10]. However, a high protein diet is also reported to induce metabolic disorders. High protein intake has the potential to stimulate hyperinsulinemia [11]. In addition, several studies have found that long-term high protein intake is associated with an increased risk of weight gain, type 2 diabetes, and overall mortality [12,13]. These disparate associations between protein intake and metabolic diseases seem to depend on the source of protein intake; for example, animal protein intake was positively associated with type 2 diabetes in a meta-analysis [14], whereas plant protein intake was negatively associated with high blood pressure in a prospective cohort study [15].

The reason for the disparate association according to protein sources can be explained by the quality of the protein and varied intake of other macronutrients. Protein quality is determined by its composition of indispensable and dispensable amino acids, which vary according to meat, and non-meat or plant-based foods. The amino acid composition may affect the digestibility of the protein [16], which can be linked to specific biological mechanisms. In addition, increasing protein intake alters the intake of other nutrients such as refined carbohydrates and saturated fat, depending on which protein sources are mainly consumed, since protein is derived from various food sources. Therefore, the intake of a particular protein source and the subsequently replaced nutrients are the major factors that explain the effects of increased protein intake on metabolism [17].

Traditional Korean diets are characterized by a high composition of various plant foods [18,19]. However, Korean diets have become westernized, particularly in the younger population [20], and the consumption of animal food products has increased [21,22,23]. The relationship between protein intake by food source and metabolic diseases among Koreans has been examined, but limited to the elderly population [24,25,26], while there are many studies including younger and older adults from other countries [14]. A Western diet has been linked to a high risk of metabolic diseases [20,27,28], and the clinical outcomes among younger adults are different from the elderly, since their dietary intakes including protein intake differ [29].

Evidence regarding the effect of protein intake by food source on the risk of metabolic syndrome for adults is required for metabolic syndrome management among Korean. To elucidate this issue, we examined the association of plant and animal protein intake and metabolic syndrome among middle-aged Korean adults using nationally representative population-based survey data.

## 2. Materials and Methods

### 2.1. Study Subjects

Data were obtained from the 2013–2018 Korea National Health and Nutrition Examination Survey (KNHANES), which is conducted annually by the Korea Centers for Disease Control and Prevention (KCDC). KNHANES consists of three examinations: a health interview, health examination, and nutrition survey. The health interview collects information on socio-demographic and health behavior variables, the health examination conducts anthropometric and blood parameter measurements, and the nutrition survey captures diet-related data, such as food and nutrient intake, food frequency, and dietary behavior [30]. All examinations are performed by trained interviewers or medical personnel with the participants’ written consent. The protocols for data collection were approved by the Institutional Review Board of the KCDC (2013-07CON-03-4C, 2013-12EXP-03-5C, and 2018-01-03-P-A).

Of 23,355 adults aged 30–64 years, subjects were excluded if their energy intake was implausible (<500 or >5000 kcal/day, *n* = 3347), if they had been diagnosed with or were being treated for hypertension, hyperlipidemia, or diabetes (*n* = 4633), if they were pregnant or lactating (*n* = 247), and if they had missing data on major variables, including 24-h dietary recall and metabolic syndrome parameters (*n* = 1643). A final total of 13,485 subjects were included (men: 5318 men; women: 8167).

### 2.2. Dietary Assessment

Dietary data, such as nutrients and food intake, were obtained using a 24-h dietary recall method by face-to-face interviews at the participant’s house. Protein intake was divided into plant or animal protein intake based on food sources. The plant protein groups included refined grains, other grains, legume and legume products, vegetables, fruits, condiments, and nuts and seeds. Refined grains included white rice, noodles, bread, and snacks. The animal protein groups included red meat, white meat, processed meat, other meat including meat by-products, fish and shellfish, processed and salted fish, eggs, and milk and dairy. The estimated energy requirement (EER) was calculated based on each participant’s sex, age, weight, height, and physical activity level according to Dietary Reference Intakes for Koreans 2015 [31]. The physical activity level of all participants was considered to be low active according to a previous study [32].

### 2.3. Definition of Metabolic Syndrome

Metabolic syndrome was diagnosed if subjects had ≥3 of the following risk factors: (1) abdominal obesity (waist circumference ≥90 cm for men and ≥85 cm for women); (2) triglyceride (TG) levels ≥150 mg/dL; (3) high-density lipoprotein cholesterol (HDL-C) levels <40 mg/dL for men and <50 mg/dL for women; (4) systolic blood pressure ≥130 mmHg or diastolic blood pressure ≥85 mmHg; and (5) fasting glucose levels ≥100 mg/dL. These standards are from the National Cholesterol Education Program Adult Treatment III [33], except for waist circumference, which was based on appropriate waist circumference threshold values for abdominal obesity in Koreans [34].

The anthropometric and clinical measurement data used for the diagnosis of metabolic syndrome were collected through the health examination by skilled clinical professional staffs with standardized protocols. The waist circumference was measured to the nearest 0.1 cm at the horizontal midpoint between the costal margin and the iliac crest. A blood sample was collected from each participant after an 8-h fast, immediately stored at 4 °C, and transported to cold storage at the Central Testing Institute in Seoul, Republic of Korea. Laboratory tests were conducted within one day after transport. The Hitachi Automatic Analyzer 7600-210 (Hitachi, Ltd., Tokyo, Japan) was used to measure TG, HDL-C, and plasma glucose levels. Blood pressure was measured three times in the right arm after the participant was seated quietly and resting for at least 5 min, using a mercury sphygmomanometer (Baumanometer^®^; Baum Co., Copiague, NY, USA). The average of the second and third measurements was used in this study.

### 2.4. Other Covariates

Socio-demographic data including age, sex, household income, and education level, and health-related lifestyle variables including drinking, smoking, and physical activity were collected through a health interview. Age groups were classified into 30–39, 40–49, and 50–64 years. Household income was categorized as the lowest, low middle, high middle, and highest. Education level was divided into ≤elementary school, middle school, high school, and ≥college/university. Among health behavior factors, drinking alcohol was divided into two groups based on the question of how often the participant had consumed alcohol over the past 1 year: ≤1/month and ≥2/month. Smoking was divided into two groups: participants were classified as a non-smoker if the participant had never smoked or had smoked in the past but did not currently smoke, and current smokers if they smoked occasionally or daily. Physical activities were evaluated with frequencies per week of high- and moderate-intensity physical activity, and walking >10 min. The categories were ≤1/week and ≥2/week depending on how frequently the participant did at least one activity of the above physical activities per week. All variables described above were used as covariates.

### 2.5. Statistical Analyses

Categorical variables of general characteristics were described as frequencies and proportions and compared by sex using the chi-square test. Continuous variables, such as energy and macronutrient intake, and protein intake from major food sources of plant and animal protein were expressed as means and their standard errors and compared by sex using Student’s *t*-test. Energy intake, and percent energy intake from carbohydrate, protein, and fat across quintiles of plant and animal protein intakes were presented as adjusted means and standard errors after adjusting for age, household income, education level, drinking, smoking, and physical activity. Odds ratios (ORs) and 95% confidence intervals (CIs) were estimated across quintiles of protein intake using the lowest quintile (Q1) as a reference by sex using multivariate logistic regression analysis to evaluate the association of plant and animal protein intakes with metabolic syndrome risk factors. In sub-analyses, the association of metabolic syndrome risk factors with animal protein intake according to levels of EER (≤EER and >EER) was assessed; ≤EER group includes participants who consume energy intake less than EER, and >EER group includes participants who consume energy intake more than EER. The *p*-value for trend across quintiles was assessed using the median protein intake (g/day) of each quintile. The factors such as age, energy intake, the percentage of energy intake from saturated fat, monounsaturated fat, and polyunsaturated fat, household income, education level, drinking, smoking, and physical activity were used as independent variables to adjust in the analysis.

All statistical analyses were performed using SAS software version 9.4 (SAS Institute, Cary, NC, USA) and the PROC SURVEY command in SAS, which incorporates a complex sampling design for a national survey. Survey sample weights, strata, and clusters were applied in the PROC SURVEY procedure.

## 3. Results

### 3.1. General Characteristics of Study Subjects

Table 1 shows the general characteristics of study subjects by sex. The proportion of women was higher than men (men: 39.4%; women: 60.6%; *p* < 0.001). Those aged 50–64 years were the highest percentage among all age groups in both men and women (men: 36.7%; women: 34.9%; *p* < 0.001). Men were more likely to have a higher household income (*p* = 0.012) and education level above college or university (*p* < 0.001) than women. Men tended to drink, smoke, and exercise more than women (*p* < 0.001). Men consumed more energy (kcal/day), carbohydrate, protein including both plant and animal protein, fat, and fatty acids (*p* < 0.001). The percent energy intake from animal protein (7.4% ± 0.1%) was higher than plant protein (6.9% ± 0.0%) in men, while that from animal protein (7.0% ± 0.1%) was lower than plant protein (7.4% ± 0.0%) in women (*p* < 0.001). However, the percent energy intake from protein was not significantly different between men and women (*p* = 0.637) and that from carbohydrate (*p* < 0.001) and fat (*p* < 0.001) including saturated fat (*p* = 0.013) and polyunsaturated fat (*p* < 0.001) was higher in women than men.

### 3.2. Major Food Sources of Plant and Animal Protein Intake

Major food sources of plant and animal protein intake of the study subjects are shown in Table 2. Among all food sources, the top protein source was refined grains for both men (19.5 ± 0.2 g/day) and women (14.3 ± 0.1 g/day), followed by red meat and fish and shellfish (men: 14.5 ± 0.3 and 9.1 ± 0.3 g/day; women: 8.7 ± 0.2 and 5.6 ± 0.2 g/day, respectively). Major food sources for plant and animal proteins were also the same between men and women. The major plant protein sources were refined grains, vegetables, and legume and legume products, and those of animal protein sources were red meat, fish and shellfish, and white meat, for both men and women. The absolute amount of protein intake from each food group, except for fruits (*p* < 0.001) and milk and dairy (*p* < 0.001), was higher in men than women.

**Figure 1:**
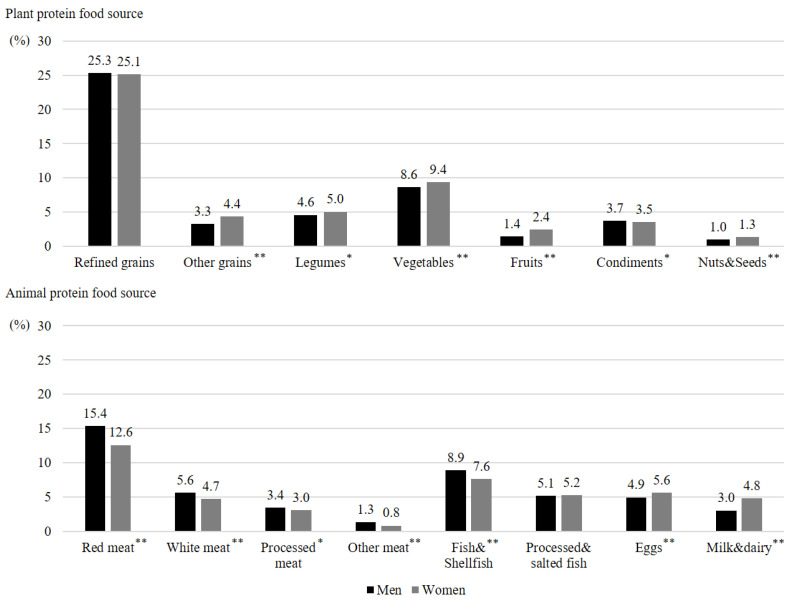
The proportion of protein intake from plant and animal food sources among total protein intake by each food group in Korean adults aged 30–64 years. *p*-values derived from Student’s *t*-test represent differences between men and women (* *p* < 0.05, ** *p* < 0.001).

Overall, the proportion of protein intake from each food group among total protein intake (protein intake from each food group/total protein intake × 100) was different between men and women by types of food sources (Figure 1). The proportion of protein intake from plant food sources was higher in women than men except for condiments (*p* < 0.05). The proportion of protein intake from refined grains was not significantly different between men and women (men: 25.3%; women: 25.1%). Among animal food sources, the proportions of protein intake from red meat (*p* < 0.001), white meat (*p* < 0.001), processed meat (*p* < 0.05), other meat (*p* < 0.001), and fish and shellfish (*p* < 0.001) were higher in men than women, while those from eggs (men: 4.9%; women: 5.6%; *p* < 0.001), and milk and dairy (men: 3.0%; women: 4.8%; *p* < 0.001) were higher in women than men. In summary, most plant food sources and several animal food sources, particularly eggs, and milk and dairy, accounted for a higher proportion of total protein intake in women than men, while meat and fish accounted for a greater proportion in men than women.

### 3.3. Energy and Nutrient Intakes According to Plant and Animal Protein Intake Levels

Energy and percent energy intakes from major nutrients according to the quintiles of intake of types of protein food sources are presented in Table 3. Energy intake increased across all types of protein intake levels in both men and women (*p* for trend < 0.001). The percent energy intake from protein and fat increased across animal protein intake quintile groups in both men and women (*p* for trend < 0.001); however, they remained almost constant regardless of incremental plant protein intake in both men and women. The percent energy intake from protein (Q5: 18.5% ± 0.2% for men, 18.7% ± 0.2% for women) and fat (Q5: 23.1% ± 0.4% for men, 24.7% ± 0.3% for women) was higher in the highest quintile group of animal protein intake than that of plant protein intake (protein Q5: 13.7% ± 0.2% for men, 14.2% ± 0.2% for women; fat Q5: 18.2% ± 0.3% for men, 19.5% ± 0.3% for women) in both men and women. Hence, the percent energy intake from carbohydrate intake decreased across animal protein intake quintile groups (*p* for trend < 0.001), while that increased across plant protein intake quintile groups in both men and women (*p* for trend < 0.001). 

### 3.4. Association of Plant and Animal Protein Intake with Metabolic Syndrome

Table 4 shows the association of protein intake by types of food sources with metabolic syndrome in Korean men and women. Positive associations of animal protein intake with abdominal obesity, reduced HDL-C, and elevated fasting glucose were observed in men. Those in the highest quintile group of animal protein intake had a higher prevalence of abdominal obesity (OR: 1.30, 95% CI: 1.00–1.70), reduced HDL-C (OR: 1.43, 95% CI: 1.07–1.90), and elevated fasting glucose (OR: 1.32, 95% CI: 1.01–1.74) than the lowest quintile group. However, the positive trend was significant only in reduced HDL-C (*p* for trend = 0.009). Men in the highest quintile group of plant protein intake had a lower prevalence of increased blood pressure compared to the lowest quintile group (OR: 0.71, 95% CI: 0.54–0.94, *p* for trend = 0.047). Neither animal nor plant protein intakes were significantly associated with any of the metabolic syndrome risk factors in women.

To confirm whether the energy intake level affects the association of animal protein intake with metabolic syndrome, the subjects were further divided by EER (Table 5). Men in the highest quintile group of animal protein intake and who had energy intake ≤ EER had an increased prevalence of abdominal obesity (OR: 1.60, 95% CI: 1.11–2.31, *p* for trend = 0.003) compared to the lowest quintile group. In addition, the association of reduced HDL-C with animal protein intake also showed a positive linear trend in ≤ EER group (*p* for trend = 0.047) in men. Animal protein intake was not significantly associated with metabolic syndrome risk factors regardless of the energy intake level in women.

## 4. Discussion

This study found that animal protein intake was positively associated with abdominal obesity, reduced HDL-C, and elevated fasting glucose regardless of fatty acid intake level in men, not in women, based on the results of data analysis of 13,485 middle-aged Korean adults who participated in the 2013–2018 KNHANES. Furthermore, stronger associations of animal protein intake with abdominal obesity were shown in men who consumed less than EER. Plant protein intake was negatively associated with increased blood pressure in men. Neither animal nor plant protein intakes were significantly associated with any of the metabolic syndrome risk factors in women.

The adverse effects of animal protein intake on abdominal obesity and cholesterol levels in men have been observed in previous studies. Animal protein intake or intakes from meat and fish were positively associated with the risk of abdominal obesity and general obesity [35,36,37] among male adults according to studies conducted in Western countries. In a Chinese longitudinal study, red meat consumption was also positively associated with an increased waist circumference in men only [38]. In a European study, high meat consumption was associated with increased cholesterol levels [39]. However, plant protein intake was inversely associated with waist circumference and weight change in the Western population [35], contrary to this study showing that plant protein intake was not associated with abdominal obesity. In summary, higher animal protein intake including meat consumption also seems to be generally associated with an increased risk of abdominal obesity and cholesterol levels in other populations; however, the effect of plant protein intake on those parameters in various populations is still inconclusive.

On the other hand, favorable effects of animal protein intake on other metabolic syndrome risk factors have been observed in several Asian populations. According to a study with rural Chinese population, increased animal protein intake was related to a significant decrease in blood pressure in women, whereas no significant association was observed for plant protein intake [40]. It has been also observed that incremental animal protein intake was more strongly related to a reduction in blood pressure than plant protein intake in Japanese adults [41]. In contrast, several Western studies have shown that animal protein intake had no significant effect on blood pressure [42,43].

One of the possible explanations for the inconsistent effects of animal protein intake on metabolic parameters in different populations might be that different proportions of animal and plant protein intake result in different diet quality. Korean men had more similar proportions of plant (51.5%) and animal (48.4%) protein intake, while women had a higher proportion of plant (54.9%) than animal (45.1%) protein intake according to this study. The high proportion of animal protein intake of Korean men found in this study is similar to the Western dietary pattern, where the percentage of protein intake from meat and dairy foods is higher (62%) than plant foods (30%) in American adults [44]. The percent energy intake from animal protein intake was also higher than that from plant protein intake in men (animal: 7.4%; plant: 6.9%), consistent with Western adult males (animal: 10.9%; plant: 6.5%) [35]. Thus, it is suggested that high animal protein intake of men in this study may result in a similar adverse effect on metabolic parameters. On contrary, the proportion of animal protein intake was very low (20%) in a Chinese rural population, and was negatively associated with a metabolic syndrome risk factor [40]. Therefore, the association between protein intakes derived from different food sources and metabolic diseases requires more attention.

Underlying mechanisms for the effect of protein intake on metabolic disorders can be explained by amino acid metabolism. For example, methionine, which is mainly from animal foods, has been shown in several in vivo studies to have atherogenic effects by inducing vitamin B deficiency, hyperhomocysteinemia, and atherosclerosis and thus is linked to an increased risk of acute coronary syndrome [45]. The other amino acids, such as glycine, tryptophan, and tyrosine, which are also abundant in animal protein, have been reported to improve blood pressure by modulating vascular tone or endothelial function, or by changing specific neuronal pathways [46,47]. Glutamic acid, which is predominantly in plants, was associated with lower arterial stiffness [48]. Thus, a different amino acid composition from plant and animal sources could play an important role in the association between animal protein and metabolic diseases and further related study are required to confirm.

Another issue is that the effects of protein intake on metabolic syndrome risk factors between younger adults and the elderly in Korea are dissimilar. Contrary to our findings, several metabolic syndrome risk factors such as abdominal obesity, hypertriglyceridemia, and high fasting blood glucose were inversely associated with protein intake among the Korean elderly [24,25,26]. According to previous studies, the elderly aged 60 years or older consumed only 30% protein from animal foods [24,29], while adults aged 30–64 years consumed about 47% protein from animal foods as observed in this study. In addition, the Korean elderly aged 60 years or older consumed about 20 g less protein per day [24,29] than adults aged 30–64 years in this study. Thus, age and energy intake as well as potential confounding variables such as physical activity, drinking, and smoking, etc. were adjusted in all analyses. A comparative analysis of the association of protein intake by food sources with metabolic diseases between younger adults and the elderly should be considered in further studies.

This study had several limitations. First, it was not able to establish a causal relationship of protein intake with metabolic syndrome due to the cross-sectional design. Second, protein intake assessed in this study could not have fully reflected the usual protein intake since we used only one 24-h dietary recall dataset. Third, this study was not able to assess the effect of variations of other macronutrient intakes such as fat and carbohydrate intake on the association of protein intake with metabolic syndrome even though the other macronutrient intake generally changes as protein intake increases. According to our findings, fat intake was positively associated with animal protein intake while carbohydrate intake was negatively associated with animal protein intake. Adjustment with fatty acid intake was conducted to attenuate the effect of fat intake on the association of animal protein intake with metabolic syndrome, however, an in-depth analysis that assesses variations in both fat and carbohydrate intakes with increments of protein intake is still required in future studies. In addition, amino acids from animal food that can influence the risk of metabolic diseases were not analyzed in the study due to lack of database, thus the interactions between animal protein or amino acid intakes on the metabolic diseases also need to be clarified in future studies. Despite these limitations, this study is the first study to analyze the association of protein intake from food sources with metabolic syndrome risk factors using a large sample of middle-aged Korean adults.

## 5. Conclusions

In summary, the animal protein intake of middle-aged Korean men was positively associated with abdominal obesity, reduced HDL-C, and elevated fasting glucose regardless of fatty acid intake level and stronger associations of animal protein intake with abdominal obesity were shown in men who consumed less than EER, while plant protein intake was negatively associated with increased blood pressure. Neither animal nor plant protein intakes were significantly associated with any of the metabolic syndrome risk factors in women. Our findings imply that lower animal protein intake may be a beneficial factor for metabolic syndrome management, particularly for abdominal obesity, reduced HDL-C, and elevated fasting glucose in middle-aged Korean men.

## Figures and Tables

**Table 1:** General characteristics of study subjects by sex.

	Men	Women	*p*-Value ^1^
***n* (%)**	5318 (39.4)	8167 (60.6)	<0.001
**Demographic factors, *n* (%)**			
Age			<0.001
30–39 years	1695 (31.9)	2476 (30.3)	
40–49 years	1673 (31.5)	2839 (34.8)	
50–64 years	1950 (36.7)	2852 (34.9)	
Household income			0.012
Lowest	358 (6.8)	653 (8.0)	
Low middle	1207 (22.8)	1940 (23.8)	
High middle	1789 (33.8)	2637 (32.4)	
Highest	1946 (36.7)	2916 (35.8)	
Education			<0.001
≤Elementary school	300 (6.2)	608 (7.9)	
Middle school	374 (7.7)	691 (9.0)	
High school	1564 (32.1)	2881 (37.4)	
≥College/University	2630 (54.0)	3531 (45.8)	
**Health behaviors, *n* (%)**			
Drinking			<0.001
≤1/month	1597 (32.3)	4304 (59.5)	
≥2/month	3345 (67.7)	2934 (40.5)	
Smoking			<0.001
Non-smoker	2930 (57.3)	7558 (94.7)	
Current smoker	2180 (42.7)	425 (5.3)	
Physical activity			<0.001
≤1/week	3190 (65.6)	5603 (72.7)	
≥2/week	1673 (34.4)	2104 (27.3)	
**Nutrient intake (mean ± SE)**			
Energy (kcal/day)	2436.9 ± 13.6	1760.6 ± 8.4	<0.001
Carbohydrate (g/day)	347.6 ± 2.0	274.4 ± 1.4	<0.001
Protein (g/day)	87.6 ± 0.6	63.3 ± 0.4	<0.001
Plant protein (g/day)	40.9 ± 0.3	32.2 ± 0.2	<0.001
Animal protein (g/day)	46.6 ± 0.6	31.2 ± 0.3	<0.001
Fat (g/day)	56.2 ± 0.5	41.7 ± 0.3	<0.001
Saturated fat (g/day)	16.8 ± 0.2	12.4 ± 0.1	<0.001
Monounsaturated fat (g/day)	18.2 ± 0.2	13.3 ± 0.1	<0.001
Polyunsaturated fat (g/day)	13.8 ± 0.1	10.5 ± 0.1	<0.001
% Energy from (%E)			
Carbohydrate	58.8 ± 0.2	63.3 ± 0.2	<0.001
Protein	14.4 ± 0.1	14.4 ± 0.1	0.637
Plant protein	6.9 ± 0.0	7.4 ± 0.0	<0.001
Animal protein	7.4 ± 0.1	7.0 ± 0.1	<0.001
Fat	20.1 ± 0.1	20.7 ± 0.1	<0.001
Saturated fat	6.0 ± 0.0	6.2 ± 0.0	0.013
Monounsaturated fat	6.5 ± 0.1	6.6 ± 0.0	0.081
Polyunsaturated fat	5.0 ± 0.0	5.2 ± 0.0	<0.001

^1^*p*-values derived from chi-squared test for categorical variables and Student’s *t*-test for continuous variables represent differences between men and women.

**Table 2:** Major food sources of plant and animal protein intake in Korean adults aged 30–64 years by sex.

	Men	Women	*p*-Value ^1^
**Plant protein sources (g/day)**			
Refined grains	19.5 ± 0.2	14.3 ± 0.1	<0.001
Other grains	2.6 ± 0.1	2.6 ± 0.1	0.887
Legume and legume products	3.8 ± 0.1	3.1 ± 0.1	<0.001
Vegetables	6.9 ± 0.1	5.5 ± 0.0	<0.001
Fruits	1.1 ± 0.0	1.4 ± 0.0	<0.001
Condiments	3.2 ± 0.1	2.2 ± 0.0	<0.001
Nuts and Seeds	0.9 ± 0.0	0.8 ± 0.0	0.708
**Animal protein sources (g/day)**			
Red meat	14.5 ± 0.3	8.7 ± 0.2	<0.001
White meat	6.7 ± 0.3	4.1 ± 0.2	<0.001
Processed meat	3.3 ± 0.2	2.0 ± 0.1	<0.001
Other meat	1.1 ± 0.1	0.5 ± 0.0	<0.001
Fish and shellfish	9.1 ± 0.3	5.6 ± 0.2	<0.001
Processed and salted fish	4.6 ± 0.1	3.4 ± 0.1	<0.001
Eggs	4.2 ± 0.1	3.5 ± 0.1	<0.001
Milk and dairy	2.5 ± 0.1	2.9 ± 0.1	<0.001

All values are presented as means ± standard errors and derived from a regression model. ^1^ *p*-values derived from Student’s *t*-test represent differences between men and women.

**Table 3:** Energy and nutrient intakes according to quintiles of plant and animal protein intake in Korean adults aged 30–64 years.

	Q1	Q2	Q3	Q4	Q5	*p* for Trend ^1^
**Men**						
**Plant protein (*n* = 5318)**	1063	1064	1064	1064	1063	
Energy (kcal/day)	1636.1 ± 28.0	2031.4 ± 27.8	2333.4 ± 27.4	2652.7 ± 29.1	3136.9 ± 30.6	<0.001
Energy from carbohydrate (%E)	57.3 ± 0.7	60.9 ± 0.6	61.9 ± 0.6	62.3 ± 0.5	63.7 ± 0.5	<0.001
Energy from protein (%E)	13.8 ± 0.2	13.7 ± 0.2	13.8 ± 0.2	13.9 ± 0.2	13.7 ± 0.2	0.901
Energy from fat (%E)	18.5 ± 0.4	18.7 ± 0.3	18.6 ± 0.3	18.6 ± 0.3	18.2 ± 0.3	0.381
**Animal protein (*n* = 5277)**	1055	1056	1055	1056	1055	
Energy (kcal/day)	1807.3 ± 30.1	2082.6 ± 27.3	2321.1 ± 30.0	2586.5 ± 29.6	3129.7 ± 33.2	<0.001
Energy from carbohydrate (%E)	69.4 ± 0.5	64.6 ± 0.5	60.6 ± 0.5	55.8 ± 0.5	48.8 ± 0.5	<0.001
Energy from protein (%E)	11.0 ± 0.1	12.7 ± 0.1	13.9 ± 0.1	15.2 ± 0.1	18.5 ± 0.2	<0.001
Energy from fat (%E)	15.5 ± 0.3	17.0 ± 0.3	19.2 ± 0.3	20.8 ± 0.3	23.1 ± 0.4	<0.001
**Women**						
**Plant protein (*n* = 8167)**	1633	1634	1633	1634	1633	
Energy (kcal/day)	1152.2 ± 18.2	1470.6 ± 19.0	1736.0 ± 18.6	1982.1 ± 20.7	2510.3 ± 24.2	<0.001
Energy from carbohydrate (%E)	59.9 ± 0.6	62.4 ± 0.5	63.3 ± 0.5	64.1 ± 0.5	63.5 ± 0.5	<0.001
Energy from protein (%E)	14.4 ± 0.2	14.2 ± 0.2	13.9 ± 0.2	13.8 ± 0.2	14.2 ± 0.2	0.148
Energy from fat (%E)	20.6 ± 0.4	19.9 ± 0.3	19.5 ± 0.4	19.3 ± 0.3	19.5 ± 0.3	0.001
**Animal protein (*n* = 8105)**	1621	1621	1621	1621	1621	
Energy (kcal/day)	1363.5 ± 22.5	1519.5 ± 24.1	1650.6 ± 23.1	1861.9 ± 24.1	2293.8 ± 27.3	<0.001
Energy from carbohydrate (%E)	71.3 ± 0.4	66.1 ± 0.4	62.6 ± 0.4	58.5 ± 0.4	51.6 ± 0.5	<0.001
Energy from protein (%E)	11.0 ± 0.1	12.6 ± 0.1	13.9 ± 0.1	15.5 ± 0.1	18.7 ± 0.2	<0.001
Energy from fat (%E)	15.2 ± 0.3	18.1 ± 0.3	19.9 ± 0.3	21.9 ± 0.3	24.7 ± 0.3	<0.001

All values are presented as means ± standard errors and derived from a multivariate regression model after adjustment for age, household income, education level, drinking, smoking, and physical activity. ^1^ *p* for trend was derived from a multivariate regression model using the median protein intake (g/day) for each quintile as the continuous variable.

**Table 4:** Odds ratios and 95% confidence intervals for metabolic syndrome parameters ^1^ according to quintiles of plant and animal protein intake in Korean adults aged 30–64 years.

	Q1	Q2	Q3	Q4	Q5	*p* for Trend ^2^
**Men**						
**Plant protein intake (*n* = 5318)**	1063	1064	1064	1064	1063	
Median intake (g/day)	22.2 ± 0.2	31.2 ± 0.1	39.0 ± 0.1	47.8 ± 0.2	62.5 ± 0.4	
Median intake (%E)	5.3 ± 0.1	6.3 ± 0.1	6.7 ± 0.1	7.3 ± 0.1	8.3 ± 0.1	
Abdominal obesity	Ref	0.88 (0.70–1.09)	0.85 (0.68–1.07)	1.04 (0.82–1.32)	1.07 (0.81–1.41)	0.313
Elevated TG	Ref	0.98 (0.80–1.20)	0.90 (0.72–1.13)	0.81 (0.63–1.04)	0.94 (0.71–1.23)	0.423
Reduced HDL-C	Ref	0.80 (0.63–1.02)	0.75 (0.58–0.96)	0.78 (0.59–1.03)	0.90 (0.66–1.24)	0.678
Increased blood pressure	Ref	0.79 (0.64–0.98)	0.75 (0.60–0.93)	0.79 (0.63–1.00)	0.71 (0.54–0.94)	0.047
Elevated fasting glucose	Ref	0.91 (0.73–1.14)	0.99 (0.79–1.24)	0.83 (0.64–1.07)	0.98 (0.75–1.30)	0.818
Metabolic syndrome	Ref	0.88 (0.70–1.11)	0.77 (0.61–0.99)	0.84 (0.65–1.10)	0.90 (0.67–1.20)	0.574
**Animal protein intake (*n* = 5277)**	1055	1056	1055	1056	1055	
Median intake (g/day)	11.4 ± 0.3	25.3 ± 0.2	37.0 ± 0.3	53.9 ± 0.3	88.4 ± 1.3	
Median intake (%E)	2.5 ± 0.1	4.9 ± 0.1	6.5 ± 0.1	8.6 ± 0.1	12.1 ± 0.2	
Abdominal obesity	Ref	1.32 (1.05–1.68)	1.15 (0.90–1.46)	1.41 (1.11–1.81)	1.30 (1.00–1.70)	0.101
Elevated TG	Ref	1.07 (0.87–1.32)	0.91 (0.73–1.13)	1.05 (0.84–1.31)	0.91 (0.71–1.17)	0.414
Reduced HDL-C	Ref	1.24 (0.98–1.56)	1.07 (0.83–1.38)	1.54 (1.19–2.00)	1.43 (1.07–1.90)	0.009
Increased blood pressure	Ref	1.02 (0.81–1.27)	0.99 (0.79–1.23)	0.97 (0.77–1.23)	0.92 (0.71–1.19)	0.576
Elevated fasting glucose	Ref	1.33 (1.07–1.64)	1.28 (1.02–1.60)	1.29 (1.02–1.64)	1.32 (1.01–1.74)	0.138
Metabolic syndrome	Ref	1.51 (1.19–1.91)	1.27 (0.99–1.64)	1.45 (1.12–1.90)	1.28 (0.96–1.71)	0.391
**Women**						
**Plant protein intake (*n* = 8167)**	1633	1634	1633	1634	1633	
Median intake (g/day)	16.4 ± 0.1	23.9 ± 0.1	30.0 ± 0.1	37.4 ± 0.1	50.5 ± 0.3	
Median intake (%E)	5.8 ± 0.1	6.8 ± 0.0	7.2 ± 0.1	7.9 ± 0.1	8.8 ± 0.1	
Abdominal obesity	Ref	0.82 (0.65–1.04)	0.85 (0.66–1.09)	1.03 (0.79–1.34)	0.89 (0.64–1.23)	0.964
Elevated TG	Ref	0.95 (0.75–1.21)	0.85 (0.66–1.10)	0.89 (0.68–1.17)	0.89 (0.64–1.23)	0.496
Reduced HDL-C	Ref	0.98 (0.80–1.19)	1.21 (0.98–1.49)	1.12 (0.91–1.38)	1.01 (0.78–1.32)	0.774
Increased blood pressure	Ref	1.03 (0.81–1.32)	0.82 (0.63–1.07)	0.77 (0.57–1.03)	0.87 (0.62–1.22)	0.229
Elevated fasting glucose	Ref	1.09 (0.88–1.35)	1.15 (0.91–1.46)	0.98 (0.77–1.26)	1.04 (0.77–1.41)	0.911
Metabolic syndrome	Ref	0.87 (0.66–1.15)	0.94 (0.70–1.27)	0.87 (0.64–1.18)	0.92 (0.62–1.35)	0.723
**Animal protein intake (*n* = 8105)**	1621	1621	1621	1621	1621	
Median intake (g/day)	7.3 ± 0.2	16.5 ± 0.1	25.3 ± 0.1	36.5 ± 0.2	61.0 ± 0.5	
Median intake (%E)	2.1 ± 0.1	4.5 ± 0.1	6.2 ± 0.1	8.1 ± 0.1	11.6 ± 0.2	
Abdominal obesity	Ref	0.77 (0.61–0.98)	0.84 (0.66–1.08)	0.83 (0.65–1.08)	0.80 (0.60–1.07)	0.301
Elevated TG	Ref	0.92 (0.73–1.15)	0.84 (0.66–1.07)	0.99 (0.77–1.27)	0.85 (0.64–1.13)	0.484
Reduced HDL-C	Ref	0.85 (0.70–1.03)	0.83 (0.68–1.00)	0.91 (0.74–1.11)	0.87 (0.70–1.09)	0.456
Increased blood pressure	Ref	0.80 (0.63–1.01)	0.86 (0.67–1.11)	0.79 (0.61–1.03)	0.83 (0.62–1.11)	0.502
Elevated fasting glucose	Ref	0.97 (0.78–1.21)	1.06 (0.84–1.33)	1.01 (0.79–1.29)	1.00 (0.76–1.31)	0.926
Metabolic syndrome	Ref	0.88 (0.67–1.15)	0.84 (0.63–1.10)	0.98 (0.74–1.30)	0.81 (0.58–1.14)	0.386

All values (ORs and 95% CIs) were derived from a multivariate logistic regression model after adjustment for age, energy intake, the percentage of energy intake from saturated fat, monounsaturated fat, and polyunsaturated fat, household income, education level, drinking, smoking, and physical activity. ^1^ Metabolic syndrome definition: (1) abdominal obesity (waist circumference ≥ 90 cm for men and ≥85 cm for women); (2) triglyceride (TG) levels ≥ 150 mg/dL; (3) high-density lipoprotein cholesterol (HDL-C) levels < 40 mg/dL for men and <50 mg/dL for women; (4) systolic blood pressure ≥ 130 mmHg or diastolic blood pressure ≥ 85 mmHg; (5) fasting glucose levels ≥ 100 mg/dL. ^2^ *p* for trend was derived from a multivariate logistic regression model using the median protein intake (g/day) for each quintile as the continuous variable.

**Table 5:** Odds ratios and 95% confidence intervals for metabolic syndrome parameters ^1^ according to quintiles of animal protein intake and the level of estimated energy requirements (EER) **^2^** in Korean men aged 30–64 years.

Animal Protein Intake	Q1	Q2	Q3	Q4	Q5	*p* for Trend ^3^
**Men (*n* = 5277)**	1055	1056	1055	1056	1055	
**≤EER (*n* = 3106)**	862	789	658	531	266	
Abdominal obesity	Ref	1.26 (0.97–1.64)	1.15 (0.56–1.53)	1.54 (1.15–2.07)	1.60 (1.11–2.31)	0.003
Elevated TG	Ref	1.09 (0.85–1.40)	0.91 (0.69–1.20)	1.00 (0.75–1.33)	0.83 (0.57–1.19)	0.333
Reduced HDL-C	Ref	1.23 (0.95–1.60)	1.17 (0.87–1.58)	1.67 (1.22–2.29)	1.26 (0.81–1.94)	0.047
Increased blood pressure	Ref	0.97 (0.75–1.27)	0.92 (0.70–1.20)	0.94 (0.69–1.28)	0.95 (0.64–1.42)	0.929
Elevated fasting glucose	Ref	1.18 (0.92–1.52)	1.22 (0.92–1.62)	1.32 (0.97–1.78)	1.28 (0.89–1.86)	0.087
Metabolic syndrome	Ref	1.43 (1.08–1.89)	1.31 (0.97–1.76)	1.66 (1.21–2.29)	1.25 (0.84–1.87)	0.082
**>EER (*n* = 2171)**	193	267	397	525	789	
Abdominal obesity	Ref	1.09 (0.60–1.99)	0.76 (0.43–1.34)	0.89 (0.51–1.54)	0.87 (0.51–1.48)	0.714
Elevated TG	Ref	0.96 (0.61–1.50)	0.91 (0.58–1.41)	1.12 (0.72–1.74)	0.97 (0.63–1.51)	0.920
Reduced HDL-C	Ref	1.36 (0.76–2.42)	0.96 (0.53–1.71)	1.54 (0.89–2.69)	1.55 (0.89–2.72)	0.057
Increased blood pressure	Ref	1.22 (0.73–2.04)	1.26 (0.77–2.06)	1.14 (0.71–1.84)	1.05 (0.66–1.67)	0.526
Elevated fasting glucose	Ref	1.94 (1.22–3.10)	1.48 (0.94–2.33)	1.43 (0.92–2.23)	1.60 (1.00–2.54)	0.423
Metabolic syndrome	Ref	1.71 (0.98–3.00)	1.19 (0.70–2.05)	1.20 (0.70–2.07)	1.24 (0.71–2.15)	0.868

All values (ORs and 95% CIs) were derived from a multivariate logistic regression model after adjustment for age, energy intake, the percentage of energy intake from saturated fat, monounsaturated fat, and polyunsaturated fat, household income, education level, drinking, smoking, and physical activity. ^1^ Metabolic syndrome definition: (1) abdominal obesity (waist circumference ≥ 90 cm for men and ≥85 cm for women); (2) triglyceride (TG) levels ≥ 150 mg/dL; (3) high-density lipoprotein cholesterol (HDL-C) levels < 40 mg/dL for men and <50 mg/dL for women; (4) systolic blood pressure ≥ 130 mmHg or diastolic blood pressure ≥ 85 mmHg; (5) fasting glucose levels ≥ 100 mg/dL. ^2^ ≤EER: energy intake less than EER; >EER: energy intake more than EER. ^3^ *p* for trend was derived from a multivariate logistic regression model using the median protein intake (g/day) for each quintile as the continuous variable.
